# Osseous Sarcoma: Is Sarcoma the New Emperor of Maladies? Case-Series with Brief Review of the Literature

**DOI:** 10.1155/2019/5602827

**Published:** 2019-05-02

**Authors:** Dikshya Sharma, Naureen Narula, Bindu Mudduluru, Bino Joseph, Dany Elsayegh

**Affiliations:** ^1^Department of Pulmonary and Critical Care, Staten Island University Hospital, Staten Island, New York 10305, USA; ^2^Department of Internal Medicine, Staten Island University Hospital, Staten Island, New York 10305, USA

## Abstract

Sarcoidosis is multisystem disease that has been amusing physicians since its discovery in 1989 by Caesar Boeck. More than 10,000 articles have been published in the literature by far, and each time it affects a new organ. Involvement of bone has been recently discovered and because it is still a rare entity, it is important to focus on the presenting systems and also to be able to differentiate it from other closely related diseases including but not limited to tumor and other infectious processes. We describe two cases of osseous sarcoma in two relatively young but surprisingly asymptomatic patients.

## 1. Case 1

**Figure 1:**
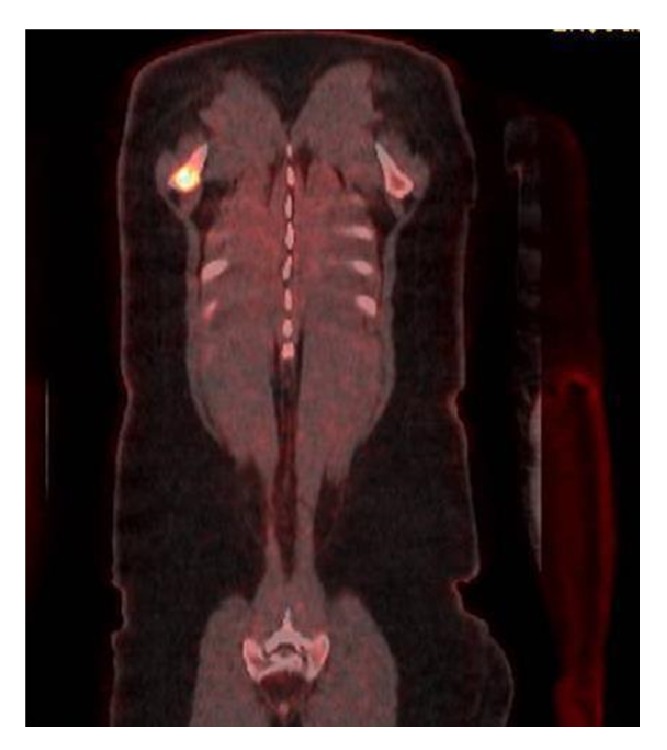
FDG avid lesion in right scapular bone.

**Figure 2:**
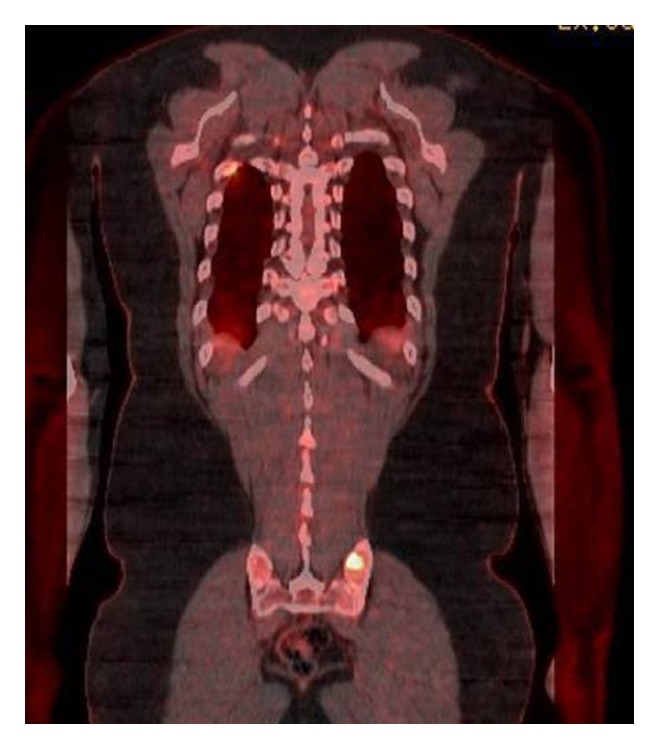
FDG avid lesion in left sacrum.

39-year-old male with no significant past medical history presented to the urgent care for some discomfort in chest. He was referred to emergency department for suspected pulmonary embolism workup. Computed tomography (CT) scan (Figure 4) of the chest with contrast revealed no pulmonary embolism. However, the striking abnormality on the CT was bilateral upper lobe mass with bilateral nodules in the perilymphatic distribution with multiple enlarged mediastinal and hilar lymphadenopathy. He was referred to a pulmonologist for outpatient workup and eventually the patient underwent a positron emission tomography (PET) scan. The PET scan showed numerous FDG avid lymph nodes in mediastinum along with bilateral lung nodules. Also present were lesions in the right scapular (Figure 1) and the left sacral bone (Figure 2).

The patient then underwent mediastinoscopy for biopsy of the lymph nodes. Review of the final pathology of the original biopsy specimen at our institution demonstrated noncaseating granulomas suggestive of sarcoidosis. However due to uncertainty of the bone lesions he was sent for biopsy of bone lesions which were reported positive for sarcoid like pattern. The patient received oral prednisone 40 mg/day therapy and subsequently had complete recovery of his symptoms. On a 3-month follow-up, repeated CT scan showed resolving mass and lymph node size.

## 2. Case 2

**Figure 3:**
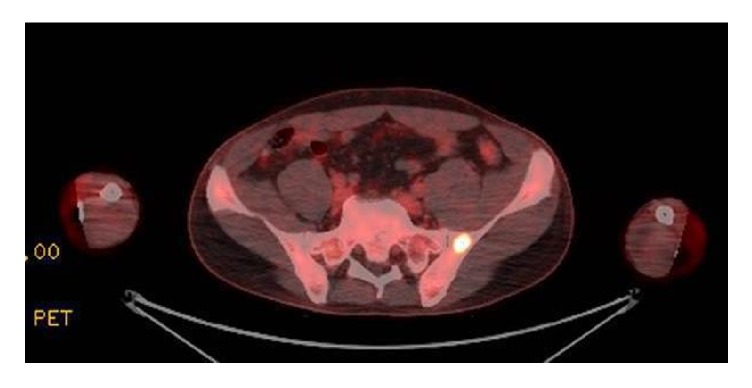
FDG avid lesion in left iliac bone.

**Figure 4:**
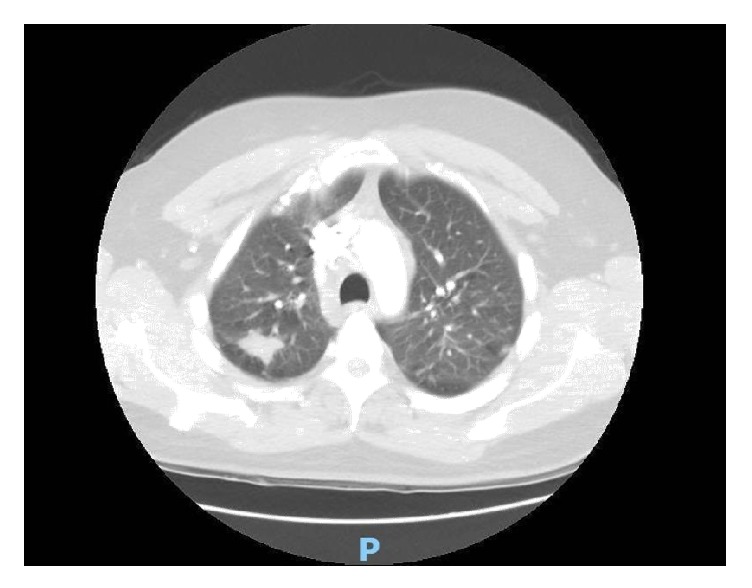
CT lung depicting right upper lobe lung nodule (Case 1).

**Figure 5:**
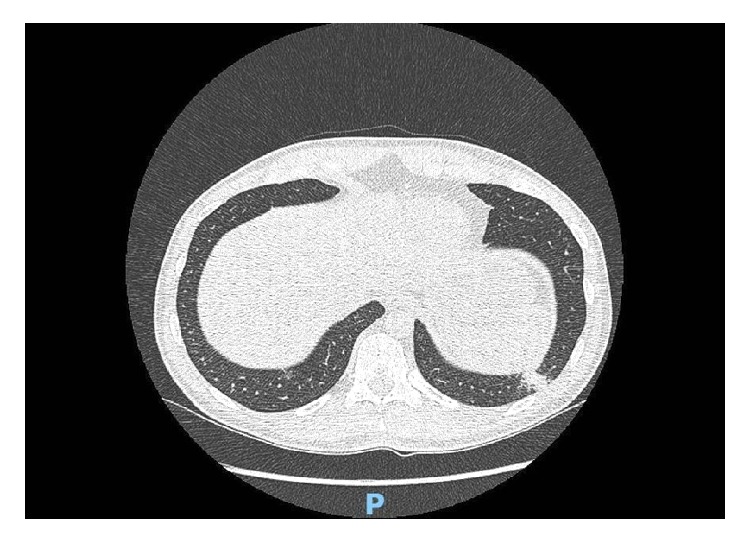
CT of chest showing lower left lobe nodule (Case 2).

38-year-old male presented to emergency department for right abdominal pain. CT abdomen showed right renal stone along with left lung lower lobe opacity. He was referred to outpatient pulmonary clinic where he was sent for CT scan chest (Figure 5) which again showed the left lower lobe opacity with mediastinal lymphadenopathy. A PET scan was done revealing multiple sites of pathologic uptake suspicious for biologic tumor activity in left lower lobe node, mediastinal lymph node, and lesion in left iliac bone (Figure 3). He was sent to a cardiothoracic surgeon wherein the patient underwent mediastinoscopic guided biopsy of the lymph nodes along with biopsy of the iliac bone lesion. Both biopsy results were positive for sarcoid like pattern. Subsequently, he also underwent left lower lobe lesion biopsy which also appeared positive for sarcoid.

## 3. Discussion

Sarcoidosis is a chronic systemic inflammatory disease characterized by infiltration of noncaseating granulomas in multiple organs and in some cases permanently impairing organ function. The epidemiology of the disease still remains poorly defined. The interplay of genetics, immune system, and environment is thought to trigger this multisystem disease in susceptible host. People of black ethnicity are more frequently involved than other races with incidence peaking in young adults [1]. It can affect any system, with lungs being involved in more than 90% of time [2]. Sarcoidosis of the bone or osseous sarcoma is relatively rare with reported incidence ranging from 3 to 13% [3]. We reviewed cases of sarcoma with bone involvement that has been reported in the literature so far. Affected patients were mostly asymptomatic. In many cases, incidental bone lesion was discovered during imaging for some other reason. Few patients presented with bony pain [4]. Hence, it can be argued that involvement of bone may be more common than reported, due to lack of symptoms and advanced imaging [5]. Small tubular bones of the appendicular skeleton were more commonly affected than the axial skeleton and it was often accompanied by overlying skin disease. Infiltration of granuloma in bones gives nonspecific osteolytic lesions on imaging. Radiographically, bone lesions were described as destructive, lytic, or permeative [6], thereby mimicking osteomyelitis, bone cysts, or neoplasms (e.g., multiple myeloma, osteoblastoma, or metastases) [4, 7]. Serum calcium and alkaline phosphatase levels were however normal despite multiple affected bones, reflecting distinct pathophysiology compared with other diseases as malignancy, Paget's, osteomalacia, or osteoporosis. [8]. Bone involvement was indicative of chronic severe sarcoidosis with multiorgan involvement. Bone biopsy was needed to confirm diagnosis as imaging alone was not sufficient to differentiate sarcoidosis from other pathologies. Bone lesions were not an indication for specific treatment unless symptomatic. There are no guidelines for the management of osseous sarcoidosis. It is evidence based and derived from extrapolations from pulmonary involvement. Corticosteroids did not improve abnormal bone architecture but was seen to decrease soft tissue swelling. [9]. In patients requiring high dose corticosteroids for prolonged periods and in corticosteroid refractory disease steroid sparing agents were added. There is no definite evidence about which sparing agent has better outcome. Methotrexate and Leflunomide were used in these cases and evidence of their use was again based on guidelines for pulmonary sarcoidosis [10]. Studies have suggested that osseous sarcoidosis often has a favorable clinical course, though further prospective research is needed for definitive conclusions [11].

We report two cases of young patients who presented for varied symptoms but were found to have PET positive lesions on their bone. Both patients had low risk for TB. Since both of the patients had biopsy proven sarcoid no other exams were sent. Due to limited knowledge about this disease entity, these patients had to undergo bone biopsy to confirm diagnosis of osseous sarcoma. Both patients did not undergo any form of treatment as they were asymptomatic. Both patients were followed in pulmonary clinic to monitor progression of disease. Till date, both patients remain clinically and radiologically stable.

## 4. Conclusion

Involvement of the bone by sarcoid is still considered relatively rare; therefor awareness of this clinical entity can help the physicians as well as radiologists to broaden the differential diagnosis of involvement of lungs and bone together, leading towards early diagnosis and prompt treatment.
